# Upskilling or deskilling? Measurable role of an AI-supported training for radiology residents: a lesson from the pandemic

**DOI:** 10.1186/s13244-024-01893-4

**Published:** 2025-01-29

**Authors:** Mattia Savardi, Alberto Signoroni, Sergio Benini, Filippo Vaccher, Matteo Alberti, Pietro Ciolli, Nunzia Di Meo, Teresa Falcone, Marco Ramanzin, Barbara Romano, Federica Sozzi, Davide Farina

**Affiliations:** 1 Department of Medical and Surgical Specialties, Radiological Sciences and Public Health, University of Brescia, Brescia, Italy; 2 Department of Information Engineering, University of Brescia, Brescia, Italy; 3 Radiology Unit 2, ASST Spedali Civili di Brescia, Brescia, Italy

**Keywords:** Artificial intelligence, Upskilling-deskilling, Radiology resident training

## Abstract

**Objectives:**

This article aims to evaluate the use and effects of an artificial intelligence system supporting a critical diagnostic task during radiology resident training, addressing a research gap in this field.

**Materials and methods:**

We involved eight residents evaluating 150 CXRs in three scenarios: no AI, on-demand AI, and integrated-AI. The considered task was the assessment of a multi-regional severity score of lung compromise in patients affected by COVID-19. The chosen artificial intelligence tool, fully integrated in the RIS/PACS, demonstrated superior performance in scoring compared to the average radiologist. Using quantitative metrics and questionnaires, we measured the ‘upskilling’ effects of using AI support and residents’ resilience to ‘deskilling,’ i.e., their ability to overcome AI errors.

**Results:**

Residents required AI in 70% of cases when left free to choose. AI support significantly reduced severity score errors and increased inter-rater agreement by 22%. Residents were resilient to AI errors above an acceptability threshold. Questionnaires indicated high tool usefulness, reliability, and explainability, with a preference for collaborative AI scenarios.

**Conclusion:**

With this work, we gathered quantitative and qualitative evidence of the beneficial use of a high-performance AI tool that is well integrated into the diagnostic workflow as a training aid for radiology residents.

**Critical relevance statement:**

Balancing educational benefits and deskilling risks is essential to exploit AI systems as effective learning tools in radiology residency programs. Our work highlights metrics for evaluating these aspects.

**Key Points:**

- Insights into AI tools’ effects in radiology resident training are lacking.
- Metrics were defined to observe residents using an AI tool in different settings.
- This approach is advisable for evaluating AI tools in radiology training.

**Graphical Abstract:**

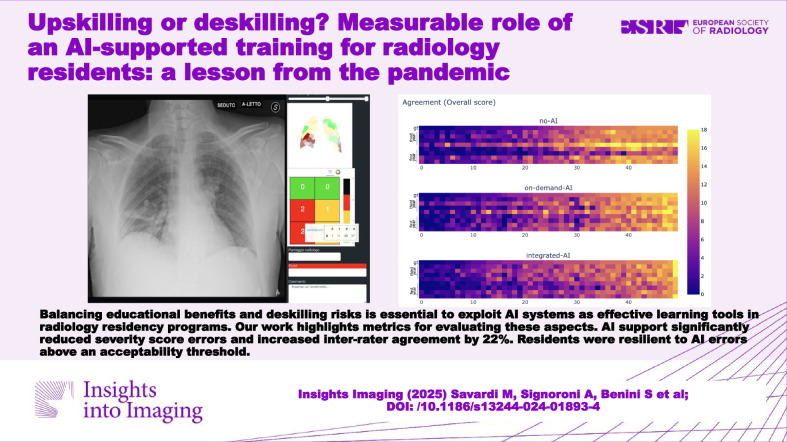

## Introduction

In the rapidly evolving landscape of artificial intelligence in medicine [1], the adoption of AI-based tools is significantly expanding in the diagnostic imaging field, where the unceasing increase in volume and complexity of medical images call for automation and advanced analytical tools [2, 3]. While AI holds immense potential to transform image-intensive healthcare departments [4], assessing its long-term benefits and risks in real-world settings remains challenging [5]. Despite positive impacts like workflow efficiency, accuracy, and healthcare cost reduction, AI-driven decision-making can lead to complex consequences [6, 7]. Challenges hindering widespread adoption include regulatory hurdles, interoperability, validation, integration complexity, data security, ethical concerns, and the potential impact on clinical decisions [8, 9]. Critical issues arising from the integration of AI into clinical workflows range from the well-known bias risk [10] to more subtle decoupled learning effects [11], occurring when newcomers to a profession learn new tasks already in symbiosis with AI tools, thus running the risk of being unprepared to handle a larger caseload from the real world. More in general, as diffusion and reliance on AI grows, there is a risk of knowledge transfer or delegation to AI systems, potentially compromising critical thinking skills [12]. Moreover, both the complacency for automation as well as the aversion to algorithms [13] can easily lead to a variety of deskilling effects [14]. To ensure that the next generation of professionals is prepared to work effectively with AI while maintaining essential skills, we must address situations where clinicians misinterpret AI outputs or respond inadequately to automation pitfalls. To mitigate all these risks, we must understand how AI systems interact with humans to develop best practices for training medical professionals in AI-integrated environments [15–17] and to guarantee safe and effective medical practice [18–20].

This article explores the potential impact of AI-assisted systems on radiology residents, assessing what can be inferred and measured with respect to the apparent clash between the phenomena of ‘upskilling’ and ‘deskilling’ [21]. Given their ongoing training, residents may be more susceptible to both positive and negative effects of AI compared to experienced radiologists [12, 22, 23]. While AI’s impact on radiology, especially residency training, is already being debated, with commentaries [24, 25], reviews [26] and survey contributions [27, 28], there is a substantial lack of quantitative studies in this field.

To address this gap, we conducted a pilot study on a cohort of residents leveraging BS-Net [29], a multi-neural network model developed to automatically assess the severity of COVID-19 pneumonia from chest radiographs (CXRs) by estimating a composite severity score [30], the assessment of which represents a challenging visual learning task for both radiologists and AI. Moreover, its multi-regional and semi-quantitative nature implies a certain degree of inter-rater variability. The suitability of selecting BS-Net for this study depends on several key factors. First, it demonstrated superior performance compared to average radiologists in a large clinical staff and a reduced inter-rater variability [29]. Second, it has been fully integrated into the hospital RIS-PACS of one of the largest hospitals in Italy, where BS-Net predictions are accessible through a dedicated RIS interface, which is integrated into the clinician’s workflow. In addition, the tool has been extensively evaluated by an interdisciplinary team of over 50 experts using a Trustworthy AI assessment methodology [31] and is currently included in the Organization for Economic Co-operation and Development (OECD) AI Observatory’s Catalogue of Tools and Metrics for Trustworthy AI [32].

To assess the possible ‘upskilling’ effect and ‘deskilling’ resilience of residents using AI, we tested three scenarios: no AI, on-demand AI, and integrated AI. Since obtaining tangible clinical benefits from AI depends on the ability of physicians to manage the delicate balance between trust and skepticism, we aim to contribute observations and evidence on this delicate matter, which also has a profound impact on the sustainability and preservation of critical health knowledge.

## Materials and methods

### AI-supported task and tool

**Fig. 1:**
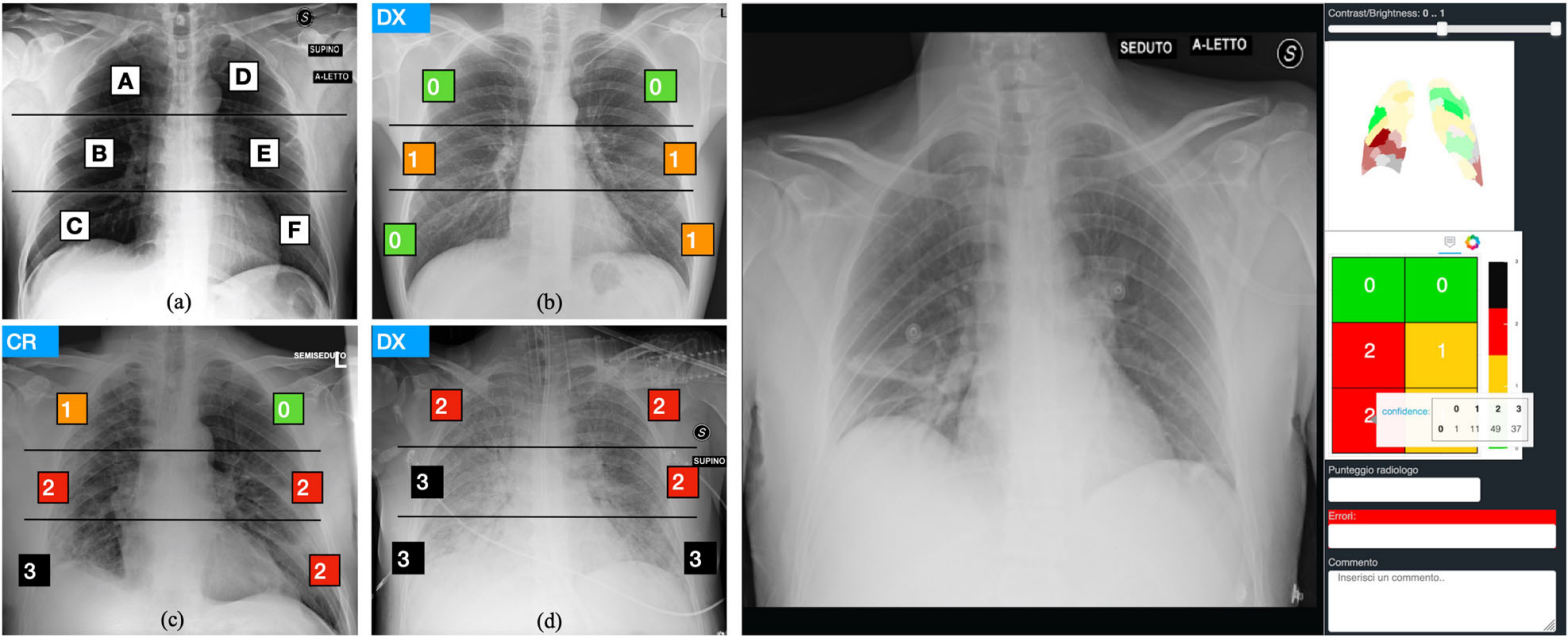
(Left) Brixia score: (**a**) zone definition and (**b**–**d**) examples of annotations. Lungs are first divided into six zones on frontal chest X-rays. Line A is drawn at the level of the inferior wall of the aortic arch. Line B is drawn at the level of the inferior wall of the right inferior pulmonary vein. A and D: upper zones; B and E: middle zones; C and F: lower zones. A score ranging from 0 (green) to 3 (black) is then assigned to each sector, based on the observed lung abnormalities. (Right) User interface: The CXR is on the left, while on the right there is a column containing the image controls (windowing) and AI support, consisting of the explainability map, the regional score with confidence levels that pop up on mouse-over, and the label boxes where the radiologist should place his/her final score

During the first pandemic outbreak, Radiology Unit 2 of Azienda Socio Sanitaria Territoriale ‘Spedali Civili di Brescia,’ one of the largest in Italy, introduced in the clinical routine a scoring system on Chest X-ray (CXR) images, referred to as the Brixia score [30], for the assessment of lung impairment in hospitalized patients with COVID-19. This is a multi-regional score, whereby the lungs are divided into six regions, and the referring radiologist assigns each region an integer score from 0 to 3, based on localized visual assessments of the severity of lung compromise, as shown in Fig. 1 (left).

Thanks to the work of the hospital’s radiology staff of around 50 radiologists, it was possible to collect a dataset of 5000 CXRs recorded in just one pandemic month, in the spring of 2020. Trained on this dataset, BS-Net has demonstrated the ability to robustly predict Brixia score values on images representative of the entire clinical reality and complexity, from different radiographic acquisition modalities (computed radiography and digital radiography) and manufacturers, acquisition directions (anteroposterior and posteroanterior) and patient conditions (e.g., standing, supine, with or without the presence of life-support systems). On a curated ‘consensus-based gold standard’ (CbGS) portion of the test dataset, rated by five radiologists, BS-Net surpassed radiologists in terms of prediction accuracy and inter-rater variability (see [29] for details).

BS-Net was also experimentally deployed on the hospital RIS/PACS, where a user interface has been designed to provide an AI-based support, to the radiologists for defining the Brixia score. This interface graphically presents confidence levels for each AI-assigned partial score in every lung region, along with an explainability map that offers insights into how BS-Net operates in such a complex task (Fig. 1, right).

### Participants and data

Leveraging the implemented version of BS-Net, we analyzed the learning effects on residents resulting from the use of this integrated AI support system in clinical activity. The participants in our study were eight radiologists in training, four of whom were in their first year and four in their third year, and with whom we wanted to explore two main aspects. First, we investigated how different levels of integration between the AI tool and residents could potentially improve their scoring performance. Second, we assessed the ability of the residents to take control in the critical event of machine failure. Specifically, we evaluated the clinical performance of the residents in three distinct scenarios:

- no-AI: the RIS/PACS interface presents only the CXR for Brixia score grading without any AI assistance;
- on-demand-AI: the interface only displays the CXR, but AI support can be accessed by simply clicking a button on the RIS. Once accessed, a panel in the RIS displays the six Brixia scores assigned to each lung region by the AI support, together with the confidence levels for each score and the corresponding explainability map;
- integrated-AI: the RIS/PACS interface simultaneously displays the CXR and all information provided by the AI support system (confidence values and map).

Each resident evaluated a set of 50 images for each scenario, covering the total of 150 images belonging to the CbGS dataset annotated by the five board-certified radiologists [29]. This dataset was partitioned into three fixed subsets, one for each scenario, so that all residents operate on the same image data. The images for each subset were randomly selected to ensure balance between variables such as age, Brixia score (global), gender and average score error by the five radiologists compared to the consensus score of the CbGS dataset, as shown in Table 1.

**Table 1:** Baseline characteristics of the three examined dataset blocks (50 images each, for a total of 150 CXR) reported in mean and standard deviation values

Scenario	no-AI	on-demand-AI	integrated-AI
Age	65.04 (19.94)	66.06 (12.61)	63.31 (13.43)
GT Brixia score (global)	6.48 (4.95)	7.36 (4.49)	7.66 (5.19)
Male sex	0.70 (0.46)	0.64 (0.48)	0.70 (0.46)
Error from 5 radiologists on CbGS	1.60 (1.53)	1.74 (1.68)	1.82 (1.69)

For this dataset, a Consensus-based Gold Standard (CbGS) was built by five board-certified radiologists

Before starting the tests, the participating radiologists received training on the AI system where they were shown examples generated by the same AI model used in the study.

### Metrics

Given the reported positive impact associated with the use of BS-Net on CXR scoring in terms of increasing inter-rater agreement (see [29]), for each scenario considered here (no-AI, on-demand-AI, integrated-AI) we first calculated the mean and standard deviation values of average scoring errors (MAE and SD) computed on the CbGS dataset. Additionally, we assessed all scenarios in terms of inter-rater agreement among the eight residents when evaluating the Brixia score on the test image set. Among the common indices of inter-rater agreement, we utilized the intraclass correlation coefficient, ICC.

To evaluate potential situations that could lead to deskilling and, in the long run, undermine the continuous development of operators’ skills, we observed residents’ behavior when AI support failed. Specifically, in the two AI-supported scenarios, we assessed whether in situations of machine failure, i.e., when the AI’s prediction error exceeded an agreed threshold of ‘acceptability,’ residents uncritically relied on the AI’s suggestions or, on the contrary, demonstrated resilience to incorrect predictions.

Five expert radiologists, each with hundreds of cases of experience with such a semi-quantitative rating system, agreed to indicate ± 0.5 as an acceptable error for each region (where the score ranges from 0 to 3) and ± 2 as an acceptable error for the global score (ranging from 0 to 18). These indications were supported by both quantitative and clinical observations. First, the ± 2 threshold value was confirmed by the numerical assessment of errors performed by the same radiologists while scoring the CbGS, as reported in Table 1. Second, this threshold was supported by the prognostic value and associated use of the score as a severity indicator, which was derived from experimental evidence and clinical observations during the initial period of its application [33].

After the experimental activity, we requested the trainee radiologists to provide their opinions and experiences through a form across four axes: agreement, usefulness, trustworthiness, and future use. The full questionnaire, which utilized a 7-point Likert scale, is presented in Table 2. Additional multiple-choice questions regarding the scoring experience are shown in Table 3.

**Table 2:** Questionnaire posed to the residents after scoring

#	Question	Score
1	How useful do you consider the information provided by the AI for your task?	(1–7)
2	How much did you trust the Brixia score proposed by the AI?	(1–7)
3	Did the explainability maps help to build trust in the AI?	(1–7)
4	Would you consult the AI’s suggestions in the future?	(1–7)

Answers were provided on a seven-point range Likert scale, for its ability to capture nuances in participant responses. Specifically, 1 = Strongly Disagree, 2 = Disagree, 3 = Partially Disagree, 4 = Neutral, 5 = Partially Agree, 6 = Agree, 7 = Strongly Agree

**Table 3:** Multiple-choice questions posed to the residents after scoring

#	Question
1	What is the main reason for me consulting the AI?
	a. Fatigue
	b. Curiosity
	c. To work faster
	d. I didn’t know where to start looking at the X-ray
	e. Other: ...
2	For which other reasons did I consult the AI?
	a. Fatigue
	b. Curiosity
	c. To work faster
	d. I didn’t know where to start looking at the X-ray
	e. Other: ...
3	In consulting the IA, what did I look at most?
	a. The six values of Brixia score
	b. The values of confidence (%) inside the colored boxes
	c. The colors and color saturation in the explainability maps
	d. Other: ...
4	Considering a clinical practice context, which mode of interaction would you prefer?
	a. No-AI
	b. On-demand AI
	c. Integrated-AI
5	Considering a clinical practice context, at what time would you prefer to have AI support?
	a. Right from the start (before formulating your score hypothesis)
	b. During the formulation of your score hypothesis
	c. After formulating your score hypothesis

In the result section, we present the results obtained in the three tested scenarios: no-AI, on-demand-AI, and integrated-AI. We first report some observations and measures concerning the use of the on-demand-AI scenario, which, unlike the other scenarios, has the peculiarity of being activated by the voluntary request of the radiologist. Then, we present measures of the potential benefits related to the active use of AI techniques by residents during their training activities in the two AI-assisted scenarios and compare them both with the no-AI case. Considering the machine failure situations in the two AI-supported scenarios, we assessed the resilience attitude of the residents with respect to the AI’s erroneous predictions. Eventually, we report the responses to questionnaires aimed at evaluating the overall scoring experience in the AI-supported scenarios.

### Statistical methods

Data analysis was conducted using Python 3.10 with the libraries statsmodels 0.14.2 and scipy 1.13.0. Descriptive statistics, including means and standard deviations, were used to summarize resident demographics and scoring characteristics. To assess the impact of AI assistance on scoring accuracy, the MAE and SD of scoring errors were computed for each scenario (no-AI, on-demand-AI, integrated-AI) using the consensus-based gold standard (CbGS) as the reference. Differences in MAE between scenarios were evaluated using paired *t*-tests, with a significance level of *p* < 0.001. The Bonferroni correction was applied to adjust the significance level to account for multiple comparisons. Inter-rater agreement among the eight residents in each scenario was quantified using the Intraclass Correlation Coefficient (ICC) [34]. Specifically, ICC-1 was employed to assess the absolute agreement among residents, assuming that they represent a random sample of residents from a larger population. ICC-1 values range from 0 to 1, with higher values indicating stronger agreement.

## Results

### Usage of on-demand-AI scenario

In the on-demand-AI scenario, it is useful to distinguish the two possible cases of requested-AI and non-requested-AI support. During the experiments, AI support was requested in 70% of the cases.

**Fig. 2:**
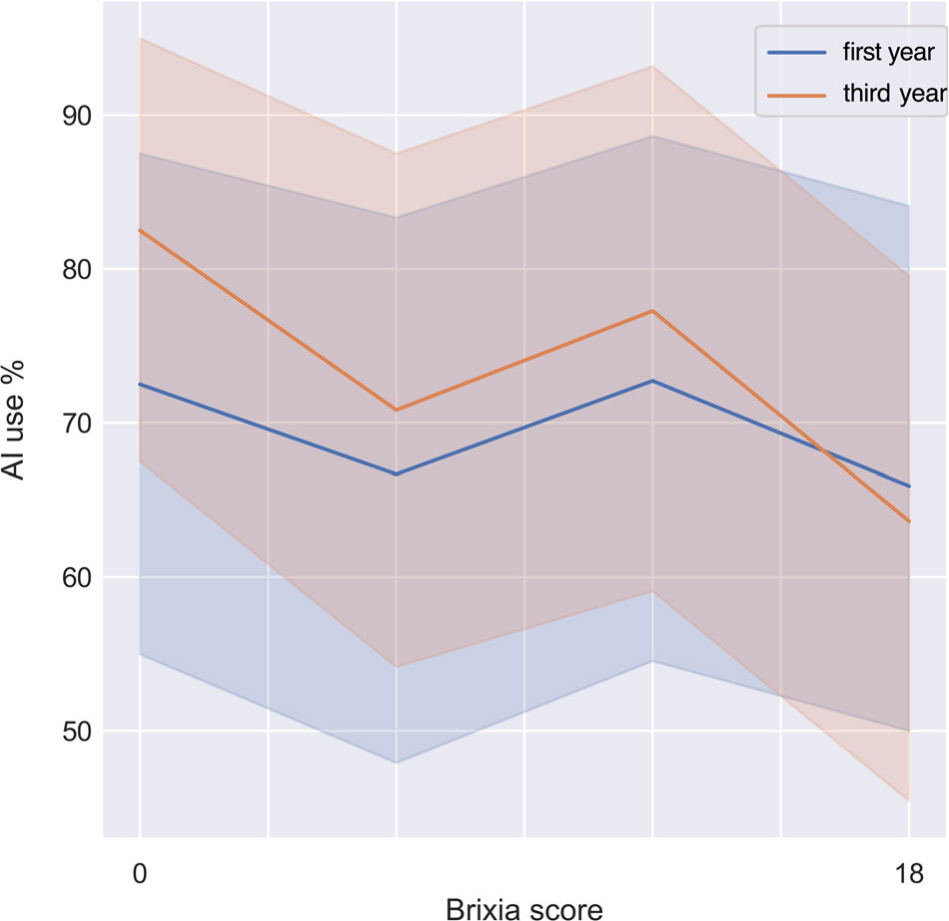
Use of AI in the on-demand-AI scenario varying the Brixia score

Regarding this requested-AI sub-scenario, further observations can be made. First, AI intervention was more frequently requested when radiologists were examining CXRs showing mild to medium levels of severity, i.e., from low to intermediate levels of the Brixia score, while less support was demanded for the most severe cases, as shown in Fig. 2.

Second, due to the relatively low number of samples for each sub-scenario, no statistically significant correlation was observed between scoring errors and the two sub-scenarios. Similarly, the distribution of errors did not show significant statistical differences between the two sub-scenarios (requested-AI vs. non-requested-AI).

### Effects on scoring agreement

**Fig. 3:**
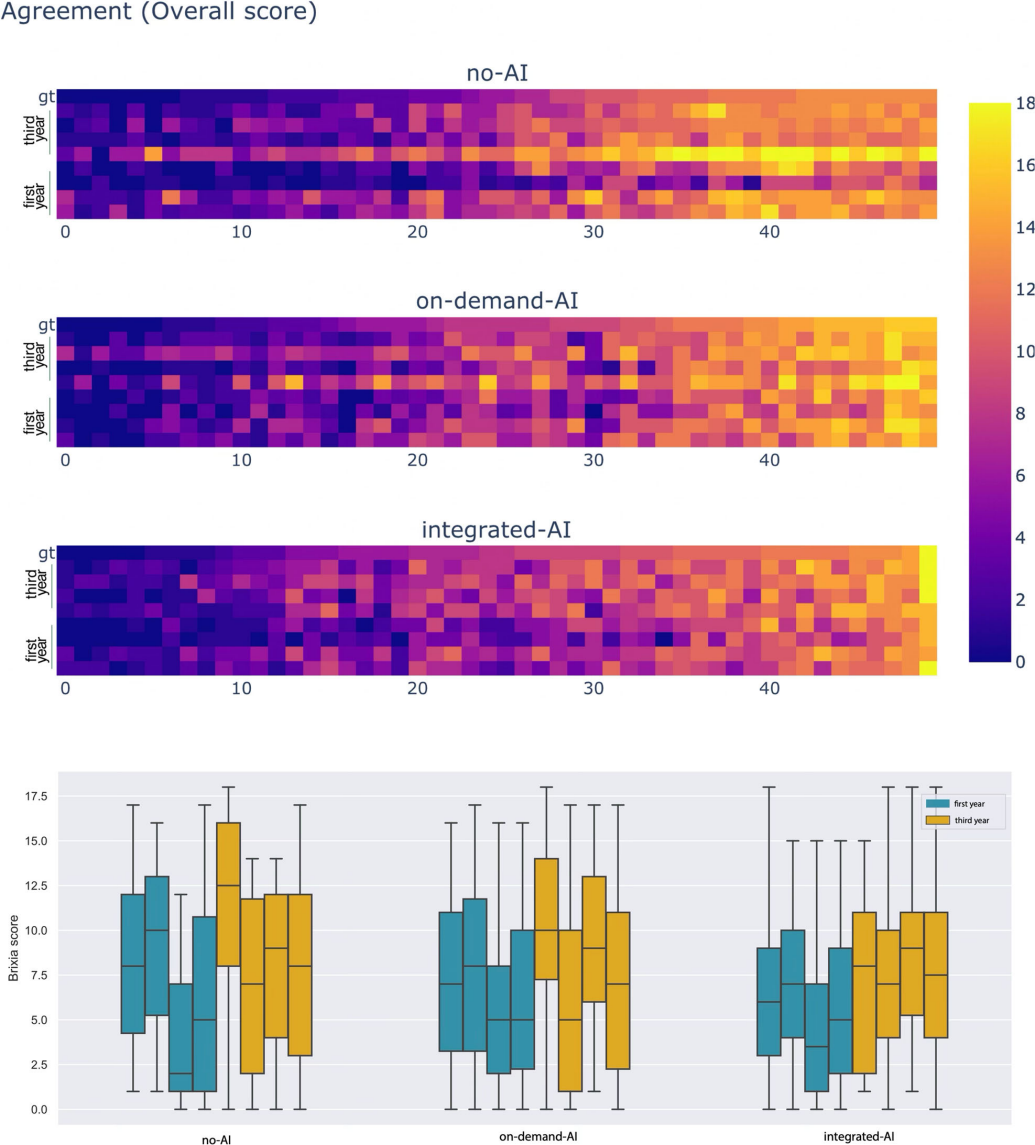
(Top) Heatmaps on scoring agreement. The heatmaps show the Brixia score of global severity assigned by the eight trainee radiologists in the three scenarios. Each column corresponds to a CXR image from the 50-image test set considered for each scenario. CXR images are ordered in ascending order (from left to right) by their consensus-based gold standard global score. (Bottom) Distributions of assigned Brixia score (global) in the three scenarios. From left to right: no-AI, on-demand-AI, and integrated-AI. Each resident is assigned the same position and color in the three scenarios

In Fig. 3 (top), we present three heatmaps, one for each scenario, to visually depict the level of agreement among the eight residents while assessing the global Brixia score on the test image set. CXR images for each scenario are ordered from left to right in increasing order of pneumonia severity, according to the consensus-based gold standard. The different global scores assigned by the eight residents are represented in the vertical dimension under the related CbGS so that higher spatial color coherence corresponds to a lower level of scoring errors.

The likely increase in the level of agreement (from no-AI to integrated-AI) is also visually depicted in Fig. 3 (bottom), where we show the distributions of the global Brixia score assigned by each resident in each scenario. As the distributions tend to align from left to right, we expect inter-rate agreement to rise accordingly.

**Fig. 4:**
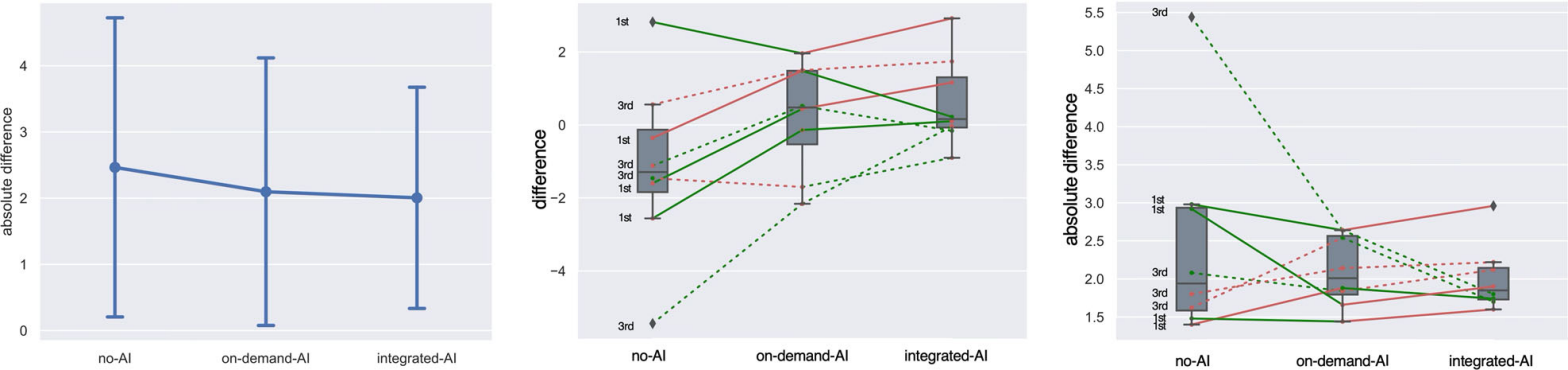
Effect of AI on trainee performance. On the left, the plot for each scenario shows the average scoring error (concerning CbGS) and its standard deviation. In the middle, the average difference between the ground truth (consensus-based gold standard) and the trainee scores on the three tests. On the right the same but with absolute mean difference. Each broken line represents a resident (dashed 3rd year; solid 1st year). A green (conv. red) line means that the error reduces (conv. increases) between the considered scenarios (from left to right)

In Fig. 4 (left), we quantitatively show how the increasing level of AI support in the three scenarios (from no-AI to integrated-AI) corresponds to a decrease in both average error and error variance, measured with respect to the consensus-based gold standard. Note that the scores referring to the intermediate scenario (on-demand-AI) accounts for both sub-scenarios: requested-AI and non-requested-AI (i.e., 100% of the cases). In particular, error differences in the two cases of no-AI vs. integrated-AI scenarios, and no-AI vs. on-demand-AI scenarios were statistically significant (*p*-value < 0.001).

The behavior of single residents in the three scoring scenarios is more evident in Fig. 4 (middle and right), where scenarios are again ordered from left to right in increasing order of AI support (no-AI, on-demand-AI, and integrated-AI), and where each broken line corresponds to a resident across all scenarios. Lines connecting two scoring scenarios are colored in green if the resident reduced the variance of his/her scoring error from the previous scenario to the following one (from left to right). Conversely, if the resident increased the variance in assigned scoring errors, the broken line is colored red.

Overall, from the performed tests, it emerged that the increasing level of AI support in the three scenarios (from no-AI to integrated-AI) correlated with an improvement of the agreement level among residents. In fact, the inter-rater correlation coefficient ICC-1 rose from 0.665 for the no-AI scenario, to 0.788 for the on-demand-AI case, and reached 0.813 in the integrated-AI one, as shown in Table 4.

**Table 4:** Inter-rater correlation coefficient ICC-1 in the three different scenarios

Scenario	no-AI [95% CI]	on-demand-AI [95% CI]	integrated-AI [95% CI]
ICC-1 coefficient	0.665 [0.57–0.76]	0.788 [0.71–0.86]	0.813 [0.74–0.87]
ICC-1 difference (from no-AI)	0.000	+0.123	+0.148

Each of the eight residents rated 50 CXR images for each scenario

Note that in the on-demand-AI scenario, the AI support was requested in 70% of cases

### Measure of resilience to machine failures

**Fig. 5:**
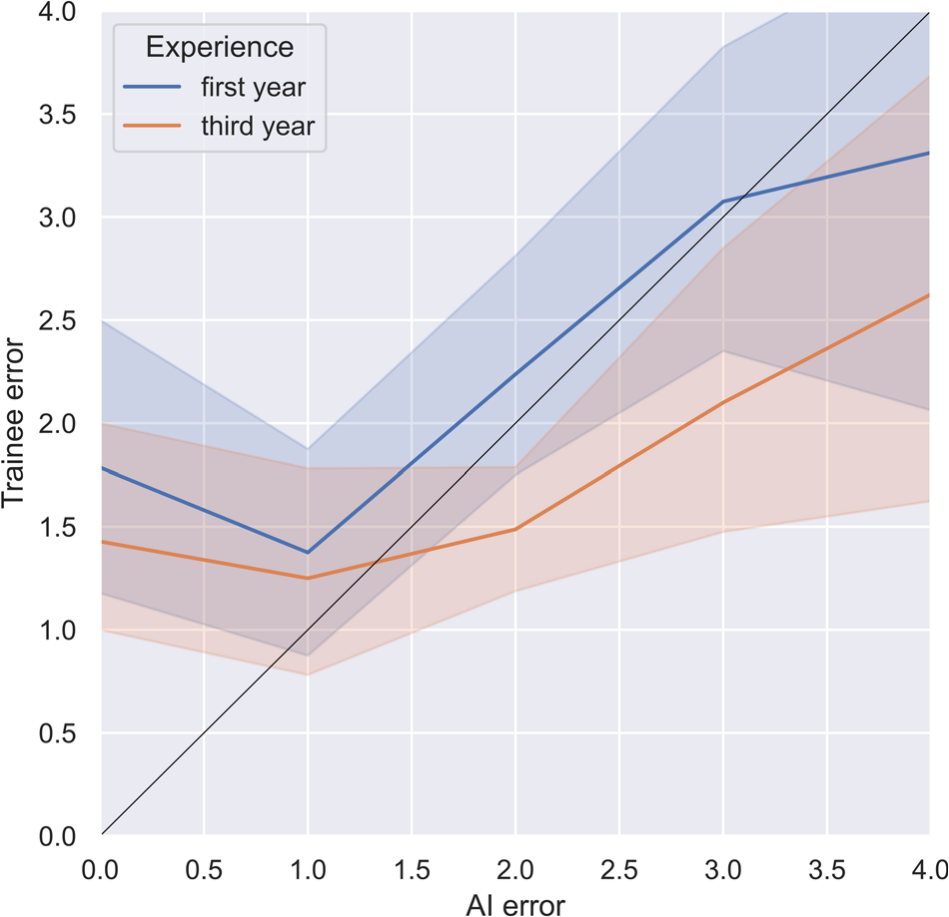
Resilience to machine failure. Relation between the (absolute) scoring error committed by the AI method (BS-Net) and the error introduced by the residents (first and third year)

In Fig. 5, we show the relation between the (absolute) scoring error committed by the AI method (BS-Net) and the error introduced by the residents, distinguishing between residents of the first from those of the third year. When the AI method introduced errors that exceeded the level of ‘acceptability’ (above 2 on the global score, as discussed in the “Metrics” section), initially the errors by residents grew with a similar trend (slope ≃ 1). However, when the neural network performed more serious errors (above 3 points), the slope of errors introduced by residents considerably diminished: the slope of third-year residents became almost flat, while first-year trainees showed a negative trend. In general, for error levels above 3, the trainees maintained an average absolute error of 2.88 in the on-demand-AI scenario and 2.75 in the integrated-AI scenario.

### Answers to questionnaires

Responses, based on a seven-point range Likert scale, showed a high perceived usefulness (average score 5.3 over 7) of the AI support, together with a moderate level of trust (average score 5 over 7). Residents also reported that the provided explanatory map helps to build trust (average score 5 over 7). In general, the trainee experience was considered positive so much so that residents look forward to using it in the future (average score 5.9 over 7).

From the answer, it emerged that the main stated motivation for using AI was to get feedback (either for curiosity, to have a second opinion, comparison or standardization), followed by compensating for fatigue. Regarding the different information provided by the AI support, the majority of the trainees mainly looked at the Brixia score and the confidence value associated with each class. Only one trainee mainly focused on the explainability map. Finally, when considering a context of use in clinical practice, the preferred mode of interaction was downstream of the formulation of the radiologist’s hypothesis of the score (such as in the on-demand-AI scenario), while no one would prefer the no-AI scenario.

Please refer to the Supplementary material for a comprehensive visualization of the responses given by the residents to questions posed in Tables 2 and 3.

## Discussion

Assessing the integration of AI-based systems into clinical practice requires thorough evaluations [35]. It also involves recognizing the importance of incorporating AI-related knowledge into radiology residency programs [28]. While the impact of AI in radiology and residency training is increasingly acknowledged [24–28], current efforts focus mainly on curricular content. The emphasis is on what to teach and how to educate physicians on machine learning, AI technologies, and their medical applications [36, 37], rather than on evaluating the practical implications of AI integration in training, such as its effects on diagnostic performance, upskilling, or mitigating the risks of deskilling in clinical practice. Quantitative studies on the effects of AI support in radiology practice have mostly focused on radiologists with prior experience [38], while there is a substantial lack of quantitative assessments in residency education contexts.

In this work, we presented a pilot study on a cohort of residents aiming at showing and experimenting how a quantitative evaluation of the impact of an AI-based support tool integrated in the clinical workflow could be conducted in a resident training context. Specifically, by exploiting BS-Net [29], an AI-based support particularly suited for estimating a clinical severity score, our study offered methodological insights and quantifiable evidence of some interesting dynamics between residency training and the use of AI support tools.

Our analysis showed a preference for on-demand AI support in 70% of cases, particularly for more severe CXRs, highlighting the adaptive nature of AI integration. Residents seemed to learn to use AI strategically, either to gather comprehensive evidence for a recommendation or to seek explanations that diverge from existing guidelines or expert knowledge [39]. However, no significant correlation was found between the rate of AI use and the rate of errors, suggesting careful and context-aware application in real clinical scenarios.

The significant reduction in relative error with increased use of AI-supported scenarios (on-demand-AI and integrated-AI) highlights AI’s crucial role in improving accuracy and reducing variability in trainee assessments [40]. The statistically significant differences in error rates between the no-AI scenario and both AI-supported scenarios further underscore AI’s positive impact on diagnostic accuracy, contributing to the discussion on optimizing radiological practice [41]. Additionally, the improvement in inter-rater agreement, with an increase in inter-rater correlation from 0.665 to 0.813 across scenarios, emphasizes the potential of AI support to enhance consistency in diagnostic assessments [29, 42] and promotes its integration into educational programs.

In cases involving certain pathologies, such as pneumothorax, effusion, and cardiomegaly, which can sometimes mislead AI interpretation, residents demonstrate a critical approach, maintaining lower average absolute errors in the on-demand-AI and integrated-AI scenarios for errors exceeding 3 points (AI vs. consensus-based gold standard). This resilience indicates that AI-assisted training not only improves accuracy but also encourages trainees to critically assess AI-generated evaluations, promoting a balanced integration of technological support and human expertise [43].

Questionnaire responses indicate a high perceived usefulness of AI, moderate levels of trust, and positive expectations for its future use. The significant role of the explanatory map in building trust highlights the importance of transparent AI decision-making processes in fostering a collaborative learning environment [44, 45]. Motivations for using AI, such as providing feedback and compensating for fatigue, align with its evolving role as a supportive tool in radiology practice, underscoring its potential to enhance the educational experience where continuous, step-by-step human support is not always feasible [46].

In a broader context, our study additionally proposes AI support as a potential solution to mitigate staff shortages in continuous resident shadowing [47]. While discussions with experienced colleagues about imaging findings and clinical-radiological correlations are crucial for developing skills and competencies, these interactions are often limited by the daily workload of understaffed teams. AI tools for image analysis can serve as complementary tutors available to residents 24/7, supplementing—rather than replacing—physical interactions when real-life constraints limit opportunities for discussion.

An in-depth exploration of AI tools’ strengths, opportunities, weaknesses, and threats is beyond this paper’s scope. However, two key considerations about AI support deserve attention: one opportunity and one threat. On the one hand, the AI tool’s image analysis, featuring explainability maps and confidence levels, aids residents by guiding severity assessments and diagnoses while indicating the AI’s confidence. This has clear educational value in training. On the other hand, relying on pre-packaged results can be comforting, especially when fatigued or handling complex cases. However, it might also lead to a satisfaction-of-search effect, potentially overlooking incidental findings that could influence the diagnosis. Over-reliance on AI could risk diminishing the diagnostic skills of both junior and senior radiologists.

Balancing the educational benefits of AI with the risk of deskilling is crucial. This requires ongoing research, ethical considerations, and regulatory frameworks to ensure AI is integrated responsibly, preserving radiologists’ core competencies. Continued research is needed to optimize AI’s role in residency programs and radiology departments, maximizing its benefits while minimizing risks.

## Supplementary information

ELECTRONIC SUPPLEMENTARY MATERIAL

## Data Availability

The 150 cases used in this study are from the BrixIA dataset (consensus subset), accessible at https://brixia.github.io/#dataset under a data research use agreement. The AI predictions for the experiment were generated by the BSnet model, available at https://github.com/BrixIA/Brixia-score-COVID-19. The clinician-AI collaboration dataset is available upon request. Data analysis was conducted using Python 3.10 with the libraries statsmodels 0.14.2 and scipy 1.13.0.

## Abbreviations

AI: Artificial intelligence
CbGS: Consensus-based gold standard
CXR: Chest X-ray
ICC: Intraclass correlation coefficient
MAE: Mean absolute error
PACS: Picture Archiving and Communication System
RIS: Radiology Information System
SD: Standard deviation

## Code availability

The analysis code is available upon request.
