# Supplementary material for: Upskilling or deskilling? Measurable role of an AI-supported training for radiology residents: a lesson from the pandemic
Source: Insights Imaging. 2025 Jan 29;16:23. doi: 10.1186/s13244-024-01893-4 (PMC11780016; doi:10.1186/s13244-024-01893-4)
Supplement: Supplementary file 1 — ELECTRONIC SUPPLEMENTARY MATERIAL [file 13244_2024_1893_MOESM1_ESM.pdf]

Upskilling or Deskilling? Measurable role of an AI-supported training for radiology residents: a lesson from the pandemic

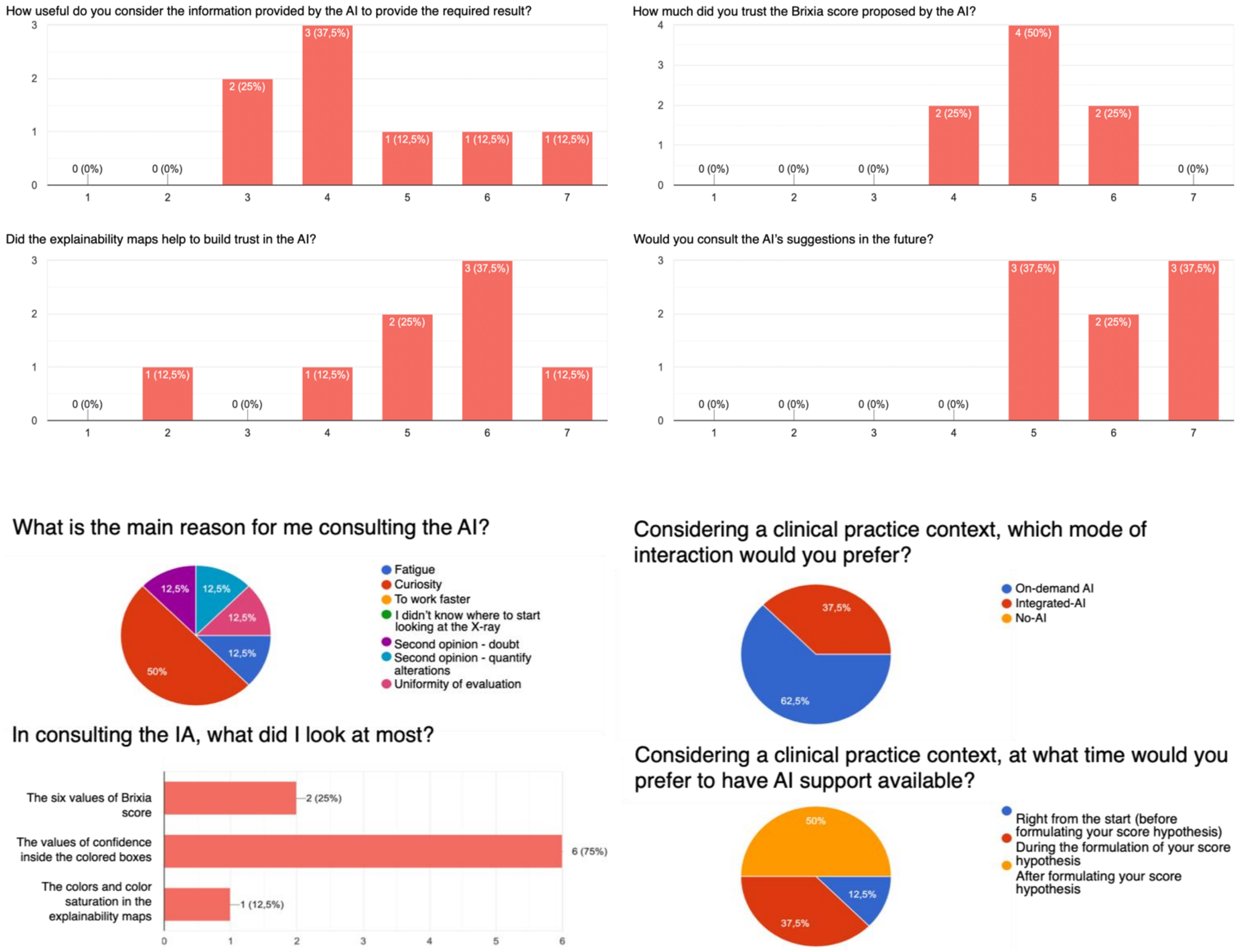

Supplementary Figure: Questionnaire on the AI-supported experience. Histograms (upper part) show the distribution of selected responses to questions posed on a seven-point range Likert scale. The diagrams (bottom part) show the distribution of selected responses to the multiple-choice questions.
